# Doxorubicin Loaded Poloxamer Thermosensitive Hydrogels: Chemical, Pharmacological and Biological Evaluation

**DOI:** 10.3390/molecules25092219

**Published:** 2020-05-08

**Authors:** Chih Kit Chung, Jomarien García-Couce, Yaima Campos, Dana Kralisch, Katja Bierau, Alan Chan, Ferry Ossendorp, Luis Javier Cruz

**Affiliations:** 1 Department of Radiology, Division Translational Nanobiomaterials and Imaging, Leiden University Medical Center, 2333 ZA Leiden, The Netherlands; c.k.chung@lumc.nl (C.K.C.); jgcouce@gmail.com (J.G.-C.); y.campos.mora@gmail.com (Y.C.); alanchan@clara.net (A.C.); 2 JeNaCell GmbH, Winzerlaer Straße 2, 07745 Jena, Germany; kralisch@jenacell.de; 3 Department of Polymeric Biomaterials, Biomaterials Center (BIOMAT), University of Havana, San Lázaro y L. Municipio, Havana 10400, Cuba; 4 Department of Ceramic and Metallic Biomaterials, Biomaterials Center (BIOMAT), University of Havana, San Lázaro y L. Municipio, Havana 10400, Cuba; 5 Department of Surgery, Leiden University Medical Center, 2333 ZA Leiden, The Netherlands; katja@bierau.net; 6 Percuros B.V., Zernikedreef 8, 2333 CL Leiden, The Netherlands; 7 Department of Immunohematology and Bloodtransfusion, Leiden University Medical Center, 2333 ZA Leiden, The Netherlands; F.A.Ossendorp@lumc.nl

**Keywords:** chemotherapy, doxorubicin, fluorescence imaging, poloxamer, sustained release, thermosensitive hydrogel

## Abstract

(1) Background: doxorubicin is a potent chemotherapeutic agent, but it has limitations regarding its side effects and therapy resistance. Hydrogels potentially deal with these problems, but several characterizations need to be optimized to better understand how hydrogel assisted chemotherapy works. Poloxamer 407 (P407) hydrogels were mixed with doxorubicin and physico-chemical, biological, and pharmacological characterizations were considered. (2) Methods: hydrogels were prepared by mixing P407 in PBS at 4 °C. Doxorubicin was added upon solutions became clear. Time-to-gelation, hydrogel morphology, and micelles were studied first. The effects of P407-doxorubicin were evaluated on MC-38 colon cancer cells. Furthermore, doxorubicin release was assessed and contrasted with non-invasive in vivo whole body fluorescence imaging. (3) Results: 25% P407 had favorable gelation properties with pore sizes of 30–180 µm. P407 micelles were approximately 5 nm in size. Doxorubicin was fully released in vitro from 25% P407 hydrogel within 120 h. Furthermore, P407 micelles strongly enhanced the anti-neoplastic effects of doxorubicin on MC-38 cells. In vivo fluorescence imaging revealed that hydrogels retained fluorescence signals at the injection site for 168 h. (4) Conclusions: non-invasive imaging showed how P407 gels retained drug at the injection site. Doxorubicin P407 micelles strongly enhanced the anti-tumor effects.

## 1. Introduction

Doxorubicin is an anti-neoplastic drug that belongs to the anthracycline family of medication. Its mode of action relies on the binding to DNA via intercalation, which results in cessation of proliferation and induction of cell apoptosis [1]. Doxorubicin has therapeutic utility for a broad range of malignancies, including solid tumors, leukemias, and lymphomas [2,3]. However, problems related to drug resistance and systemic side effects, in particular, cardiotoxicity, can substantially narrow the application of doxorubicin [4]. Thus, these drawbacks warrant, in particular, the optimization of doxorubicin delivery.

Injectable in situ gelling systems that are based on biodegradable polymers represent promising drug delivery platforms for coping with these hurdles. Owing to their promising effects on sustained drug release, various types of polymeric hydrogels have gained considerable interest for the optimization of drug delivery [5,6,7,8,9]. Poloxamer 407 (P407), trade name Pluronic 127^®^ (PF127), is a co-polymer that is based on poly (ethylene oxide)–poly (propylene oxide)–poly (ethylene oxide) triblock copolymers (EO-PO-EO) and it has found interesting applications within hydrogel drug delivery research. When sufficiently concentrated and upon temperature increase, P407 solutions become a semi-solid gel depot. P407 injectable hydrogels bear various favorable features as delivery system, including biocompatibility, non-invasive and easy administration, promotion of sustained drug release, and increasing therapeutic efficacy [10,11].

Regarding the large set of parameters that needs satisfaction, alongside the different effects that hydrogels could exert, the design and tailoring of hydrogel systems to improve cancer therapy can be a cumbersome task. Furthermore, certain characterizations will still need optimization. Drug release is a main parameter of investigation, because slow release is beneficial to keeping systemic drug levels low, whilst it allows for prolonged acting of the drug at the desired tissues [12,13,14]. In cancer drug delivery research, it is especially interesting to link release data with in vivo drug retention data, since it could provide information where the drug might sequester after injection [15,16]. Parallel to the growing interests for slow release systems, an increasing number of studies has underscored the promises of local cancer therapy [17]. As such, it is of great interest to carefully monitor in vivo drug retention. In this pursuit, researchers have deployed interesting imaging methods to track the drug delivery through the body. One example is to open up the skin where the gel was injected and measure the fluorescence from the injection depot [18]. Another conventional method is to excise tumors (into which the drug was injected) and measure the drug concentration or fluorescence intensity ex vivo [19,20,21]. However, these methods could be cumbersome due to the labor intensive and invasive procedure, since, for every measurement time-point, mice will need to be operated or sacrificed. Non-invasive fluorescence imaging is a convenient tool with a variety of applications within oncological research [22]. This technique is, for instance, well-suited to monitor locally injected drug depots (e.g., under the skin) during a longer period without the need to operate. Still, limited studies have followed the drug retention of locally injected P407 depots utilizing non-invasive imaging techniques.

Besides focusing on these pharmacokinetic parameters, it is interesting to assess how the hydrogel materials directly interact with cancer cells. When temperature and concentration increase, the micellization of the EO-PO-EO blocks ensues, which represents the initial step of gelation. Eventually, the dehydration of the inner PO core and hydration (swelling) of the outer EO shell will result in the ordered packing of the micelles, leading to the gelation of the solution [10]. This gelation process is reversible when the temperature decreases or the gel is diluted. Gel dilution and breakdown occur when the depot comes in contact with physiological fluids, with the depot slowly degrading into smaller micelle aggregates, single micelles, or EO-PO-EO chains.

Micelle drug complexes are of great interest, since they might sensitize cancer cells to chemotherapy treatment. Cancer cells can actively pump out chemotherapeutic drugs, which is one of the underlying mechanisms of drug resistance [23,24]. Micelle-drug complexes are supposedly large enough to prevent the active drug from being pumped out; therefore, they represent an appealing solution to deal with drug resistance. Until now, large hybrid micelles coupled with chemotherapeutics, in particular PF127/PF105 and PF127/PF123, have been demonstrated to be more toxic to cancer cells [11]. We were interested to evaluate whether these could promote cytotoxic effects in cancer cells that often develop resistance mechanisms, such as colon cancer cells, since the P407 hydrogels are made up of identical and smaller micelles (PF127) [25]. This evaluation provides clues of how P407 hydrogels might interact with cancer cells.

In this multiparameter study, we aim to optimize various characterizations for improved understanding of P407 hydrogel doxorubicin therapy. The main interest was to contrast P407 doxorubicin release data with non-invasive in vivo fluorescence imaging. We also focus on the interaction of P407 with cancer cells. First, time-to-gelation measurements for differently concentrated P407 hydrogels were performed in order to select an optimal formulation. In vitro cell killing assays were performed in order to assess the potential of P407-doxorubicin to kill MC-38 colon cancer cells. Next, P407 breakdown and doxorubicin release rates were evaluated. Indocyanine green (ICG) was deployed as non-toxic model drug for doxorubicin to visualize in vivo drug release from the hydrogel depot. ICG loaded hydrogels were administered in mice and fluorescence signals were recorded over time. The critical appraisal of the obtained information leads to better understanding of the different mechanisms underlying the successes of hydrogel based cancer therapy. These understandings can then be leveraged for the further fine-tuning of hydrogel therapies.

## 2. Results

### 2.1. P407 Hydrogel Time-to-Gelation Assay

**Figure 1:**
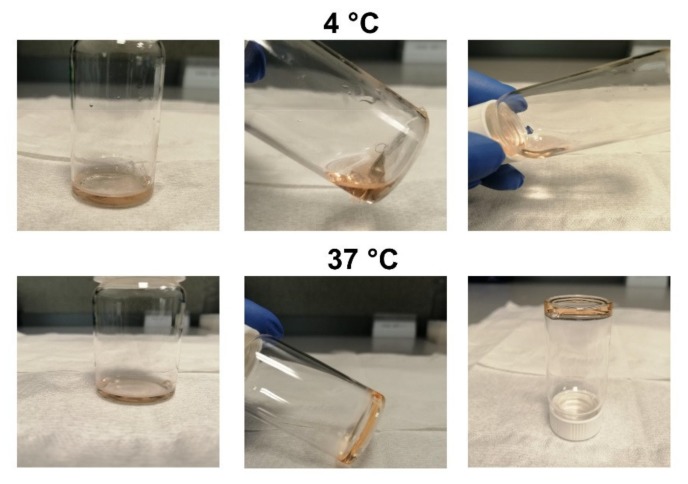
Doxorubicin loaded P407 hydrogels flow freely at 4 °C, but are solid at 37 °C.

P407 hydrogels appear as fluid when kept below the gelation temperature. Hydrogels can flow and be injected when kept below the critical gelation temperature, but they start to solidify when temperature is increased (Figure 1). The time that it takes for hydrogels to convert into the solid state is referred to as time-to-gelation, which is among others dependent on P407 concentration [10].

Different concentrations of P407 hydrogel (16, 20, 25, and 30%) were prepared and time-to-gelation measurements were performed at room temperature (RT) and 37 °C (Table 1). Based on the measurements at 37 °C, 16% P407 was excluded for further analysis, because this formulation failed to establish a solid gel depot. A large drawback would be the rapid spread of doxorubicin upon injection in vivo and, consequently, the induction of systemic side effects. At RT, the time-to-gelation was 275 ± 5 s and 133 ± 7.5 s for 25 and 30% P407, respectively. Based on this result, we considered the 25% formulation as most optimal, due to the relative slow time-to-gelation at RT, but quick gelation time of 30 ± 1 s at 37 °C. This enables easy injection at RT and prevents, in particular, the clogging of injection needles, whilst gel depots are established quickly in the body. Henceforth, the 25% P407 formulation was used for the subsequent analyses. The addition of doxorubicin (50 µg/mL) in P407 does not affect time-to-gelation at RT (264 ± 1.5) and 37 °C (29 ± 1.5 s), as shown with the 25% P407 formulation.

### 2.2. Scanning Electron Microscopy (SEM) Reveals Porous and Tunnel like Morphology of Lyophilized P407 Hydrogel

**Figure 2:**
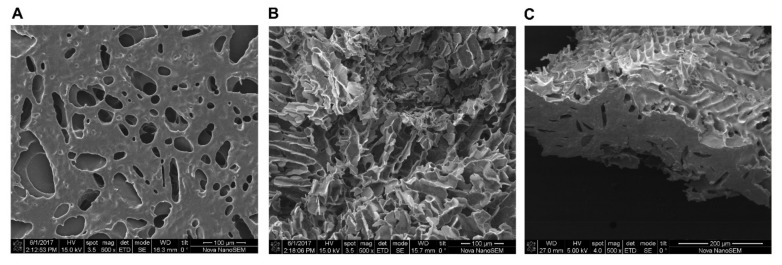
Scanning electron microscope (SEM) images showing a cross section of the 25% P407 formulation ((**A**); magnification 500×) and transversal sections ((**B**,**C**) panel; 500×).

Cross section pictures obtained by SEM microscopy revealed the typical porous structure of lyophilized P407 hydrogel specimens (Figure 2). Pores were predominantly round and oval, with pore sizes ranging from circa 30 to 180 µm, as analyzed by ImageJ software. When cut more transversally, the tunnels and channels running through the hydrogel are clearly visible, as shown in Figure 2B,C. These channels are formed between the micelle clusters when gelation takes place. Supposedly, these pores drive the release of incorporated drugs, which is, in particular, the case when drug release is driven by diffusion. Micelles might be more or less densely clustered together, depending on the P407 concentrations. Since the pore size is determined by the space between the packed micelles, the increase of P407 concentration results in smaller pores and supposedly slower drug diffusion rates [26].

### 2.3. 25% P407 Hydrogel Facilitates Doxorubicin Release Over 120 h

**Figure 3:**
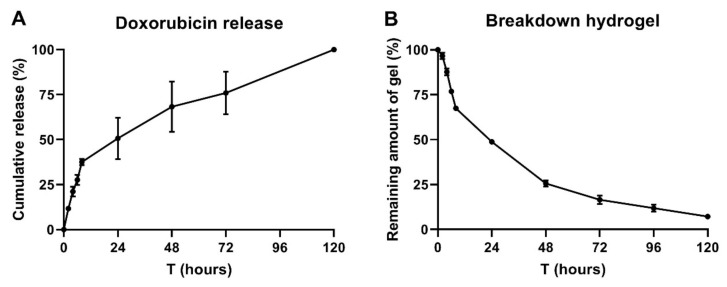
Doxorubicin release from 25% P407 hydrogel formulation. (**A**) 1 mL of hydrogel was loaded with 50 µg of doxorubicin and 4 mL PBS pH 7.4 was layered on top to serve as release medium. At regular intervals, 1 mL release medium was refreshed and doxorubicin concentrations were measured with the Cytation 5 device at 20 °C. (**B**) 25% P407 hydrogel breakdown shown in percentages. Data are shown as mean ± SD for a triplicate measurement.

**Figure A1:**
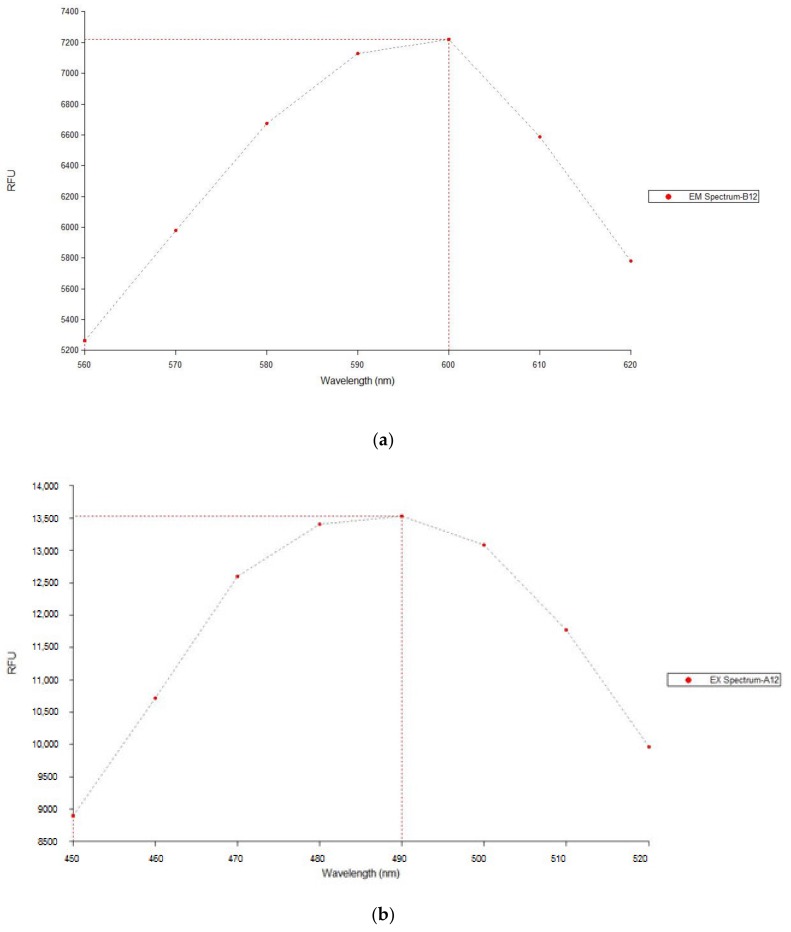
Doxorubicin emission (**a**) and excitation spectrum (**b**) determined by Cytation 5 Manager.

The ′membrane-less′ method [27,28] was considered to evaluate doxorubicin release from the 25% P407 hydrogel. In PBS, doxorubicin has an excitation peak at 595 nm upon excitation at 470 nm [29], which we confirmed with Cytation 5 measurements (Appendix A: Figure A1). A bi-phasic release pattern can be observed, as is apparent from Figure 3A. Approximately 35% of the initial doxorubicin load was released within the first 8 h, whilst the remaining 65% was more gradually released within 120 h. 50% of the loaded doxorubicin was released shortly after 24 h. Figure 3B shows the hydrogel degradation. 50% of the hydrogel was degraded after approximately 24 h, with the remaining amount being degraded after 120 h. These figures demonstrate that doxorubicin release was associated with the hydrogel breakdown rate. Hydrogel breakdown was notably pronounced during the first 8 h, because the sampling frequency was higher. This is due to the refreshment and addition of fresh release medium, where the process can cause gels to break down more quickly. Consequently, doxorubicin release rate was the quickest during the first 8 h. As the sampling frequency was reduced after 24 h, the breakdown and thus release curves became less steep. Even though hydrophilic small drug molecules, such as doxorubicin, can diffuse through the hydrogel channels, it could be contemplated that doxorubicin release was predominantly driven by hydrogel dissolution.

### 2.4. Dynamic Light Scattering (DLS) Measurements on Different Concentrations P407

**Figure 4:**
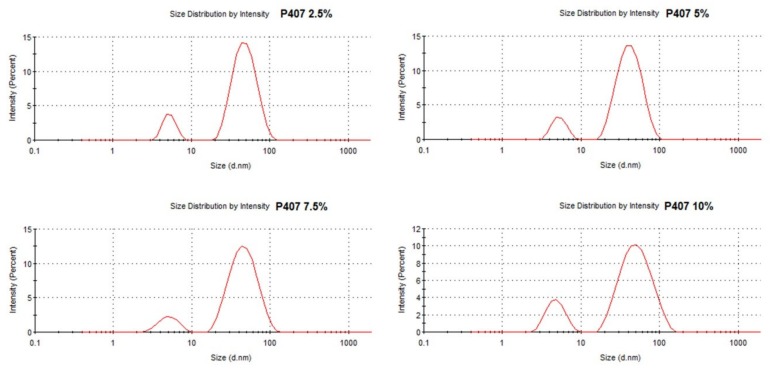
DLS measurements with representative histograms with the peak size distribution for 2.5%, 5.0%, 7.5%, and 10.0% P407.

DLS measurements were performed with 2.5, 5.0, 7.5, and 10.0% P407 formulations (prepared in MilliQ H_2_O) in order to characterize the micelles. In Figure 4, representative histograms for the size peaks are depicted for the different P407 concentrations. Table 2 summarizes the polydispersity index (PDI; which measures the heterogeneity of the particles in a solution) measurements. Micelles were already detectable at a concentration of 2.5% P407, with two size peaks being apparent in the histograms. The first peak at 5 nm represents the micelles population, whilst the second (at 50 nm) could be ascribed to aggregates of micelles. Thus, the P407 solutions appear as a non-homogenous solution of micelles and aggregates, as reported before [30]. The PDI values were comparable for all concentrations between 2.5 and 7.5% P407 (Table 2). Interestingly, the PDI increased up to 0.597 at 10.0% P407, with a higher intensity of the 5 nm peak. This indicates that, starting from 10.0% P407, the number of micelles and, thus, the ratio of micelles to aggregates is considerably increasing, which causes the corresponding rise in PDI.

### 2.5. P407 Synergize with Doxorubicin in Killing Tumor Cells

**Figure 5:**
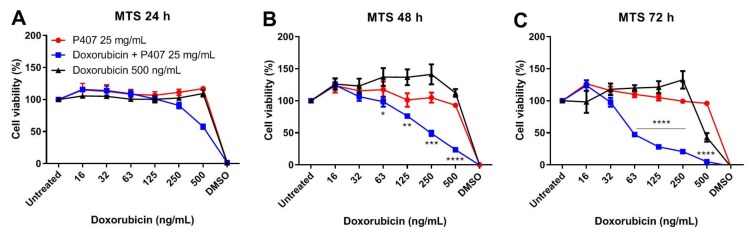
MC-38 tumor cells were treated with P407, doxorubicin or P407 mixed with doxorubicin. Both compounds were tested as a serial dilution with doxorubicin starting at 500 ng/mL and P407 starting at 25 mg/mL. After 24 h (**A**), 48 h (**B**), and 72 h (**C**) of incubation, cell viability was measured with MTS assay. Data are shown as mean ± SD for a triplicate measurement and differences were assessed using a Student′s unpaired *t*-test. * *p* < 0.05, ** *p* < 0.01, *** *p* < 0.001 and **** *p* < 0.0001 for doxorubicin vs. doxorubicin + P407.

The effect of P407 + doxorubicin on tumor cells was evaluated with an MTS (CellTiter 96^®^ AQueous One Solution Cell Proliferation) assay. It is possible that P407 (PF127) micelle-doxorubicin complexes exert stronger cytotoxic effects on tumor cells, depending on the tumor cell line. Here, P407, doxorubicin, and P407 plus doxorubicin were tested on MC-38 tumor cells for 24, 48, and 72 h. All of the compounds were titrated down starting at 25 mg/mL for P407 and 500 ng/mL for doxorubicin. P407 solution did not affect cell viability at any concentration, as could be seen in Figure 5. Furthermore, doxorubicin alone did not exert tumoricidal effects on MC-38 cells, except after 72 h incubation at 500 ng/mL (c). However, a combination of P407 and doxorubicin drastically reduced tumor cell proliferation already at low doxorubicin concentrations, notably after 48 h (Figure 5B) and 72 h (Figure 5C). Thus, these data indicate that a combination of P407 and doxorubicin facilitated the synergistic killing of MC-38 tumor cells. This is a promising result, because it shows that cell lines that are poorly responsive to doxorubicin might be sensitized with P407.

### 2.6. Hydrogel Breakdown and ICG Release Visualized by Non-Invasive Molecular Imaging

Visualizing P407 hydrogel breakdown and drug release with non-invasive fluorescence imaging could provide interesting information that might not be solely obtained from release studies. An advantage of non-invasive fluorescence imaging is that it allows for the continuous monitoring of, for instance, drug depots without the need to operate or sacrifice mice.

**Figure 6:**
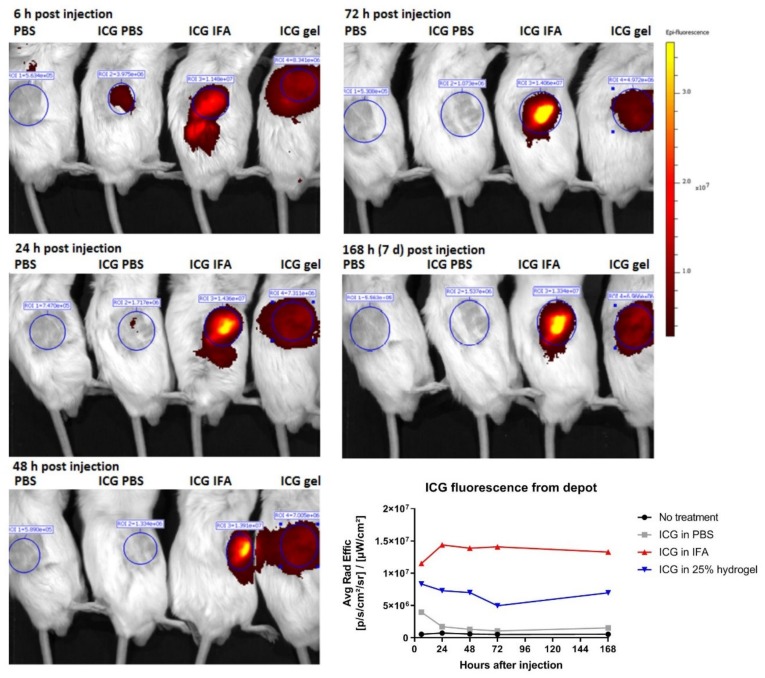
Indocyanine green (ICG) was loaded into PBS, IFA, or 25% hydrogels and administered subcutaneously in the right flank of BALB/c mice. At the indicated time-points, mice were imaged in order to track the changes of ICG fluorescence (emission peak at 810 nm). ICG in IFA was used as positive control for slow release.

Indocyanine green (ICG) was deployed as non-toxic model drug to visualize fluorescence retention in vivo. With comparable solubility in water and molar weights (ICG 774.96 g/mol; doxorubicin 543.52 g/mol), ICG was considered to bear comparable release kinetics as doxorubicin. Therefore, the use of ICG for simulation of doxorubicin release is justified. Tumor free BALB/c mice were injected with ICG in PBS, in IFA (Incomplete Freund′s adjuvant; a slow release formulation based on water-in-oil emulsion [31]) or 25% P407. 6 h, 24 h, 48 h, 72 h, and 168 h after injection, mice were imaged with IVIS. Figure 6 shows that 6 h after injection, only a minority of the ICG signal remained at the site of injection for the mouse injected with ICG in PBS (free delivery). After 24 h, the signal was clearly diminished, indicating the rapid spread of ICG when given in PBS. In stark contrast, the mouse receiving ICG in hydrogel retained the ICG signal up to 168 h (7 days). This is an indication that the ICG signal is retained at the site of injection for at least a period of 168 h. Apparently, the ICG signal that was derived from the IFA depot was almost two-fold higher than that of the hydrogel. It could not be ruled out that IFA was more potent in retaining the fluorescence signal, since it is established as a slow release system for vaccination compounds. In our previous study, it which we studied antibody release from IFA and P407 hydrogels, IFA also showed slower in vivo release kinetics, as determined by serum analysis [32]. However, imaging studies are also prone to distortion that is caused by external factors, like the fur or skin. Thus, it is possible that the oil in IFA scattered or distorted the ICG signal, thereby yielding amplified signals. For imaging studies, it is therefore essential to assure that chemical modifications applied to hydrogels do not interfere with imaging agents. While using non-invasive imaging, we thus showed how P407 hydrogels sustained small molecule drug release for a prolonged period of time at the site of injection.

## 3. Discussion

Doxorubicin is a potent anti-cancer drug. However, its application in the clinic is confronting several drawbacks, including, in particular, drug resistance and off target toxicity. Thus, these problems provide strong rationale to optimize anti-cancer drug delivery with, for instance, drug delivery systems. However, the tailoring of drug delivery systems to reduce side effects without compromising therapeutic efficacy could be a complicated task. This is, among others, owing to the large set of parameters that needs to be satisfied, considered, or is even elusive. Here, P407 hydrogels incorporating doxorubicin were prepared and pharmaceutical, biological, and physico-chemical parameters were studied. Specifically, we focused on non-invasive fluorescence imaging, which is a convenient tool to visualize drug release. Moreover, the potential of hydrogel-doxorubicin systems to confer stronger tumoricidal effects was discussed. These assessments provided profound insights regarding how hydrogel cancer therapies act, which might be especially useful to speculate regarding possibilities to further finetune hydrogel therapy.

We first evaluated the gelation time of various concentrated gels and demonstrated how these measurements provide information regarding the injectability of hydrogels, as well as the reduction of drug spread in vivo. A formulation that quickly solidifies in vivo, but slowly at room temperature, offers good injectability and reduction of drug spread in vivo. A possible undesired effect would be the drastic reduction of gelation time upon increasing P407 concentrations beyond 30%, making injection difficult. This problem can be circumvented by adding P188, which can modify hydrogel liquid to solid transition temperature [33]. Thus, highly concentrated hydrogels with P407 concentrations >30% may be prepared easily in this way; however, the effect of P188 on gel stability and burst release must be evaluated. Furthermore, hydrogels might be strengthened with crosslinkers [34]. However, the applicability and safety of crosslinkers need to be scrutinized carefully, since crosslinkers might be toxic and compromise injectability of hydrogels.

In vitro, a mix of doxorubicin and P407 induced synergistic killing of MC-38 colon cancer cells, whereas MC-38 cells were not sensitive to doxorubicin treatment alone. The strong tumoricidal effects might be ascribed to the formation of doxorubicin P407 (PF127) micelle complexes. These are formed upon dissolving chemotherapeutic drugs in a poloxamer solution higher than the critical micellization concentration [10]. Moreover, doxorubicin might be liberated as micelle complexes upon hydrogel erosion in aqueous environments. DLS measurements on P407 solutions revealed two distinctive peaks, which were already detectable at 2.5% P407. The first peak had a mean size of approximately 5 nm and it is caused by the individual micelles, whilst the second peak (50 nm) is associated with aggregates. The DLS size histograms were comparable for solutions between 2.5% and 7.5% P407, with PDIs around 0.35. When increasing the P407 concentration to 10.0%, the intensity of the left peak was clearly increasing, which was associated with an increase of PDI to 0.59. Our observations with the double peaks and PDI increase corroborate the data from a study conducted by Suksiriworapong et al. [35], in which the authors performed DLS measurements on diazepam loaded PF127 micelles.

These micelles might be protected from being pumped out from cancer cells via the glycoprotein P (P-gp) pumps, unlike chemotherapeutics that are administered in free form [23,24]. It is conceivable that doxorubicin molecules are attached to the outer shells of the micelle complexes due to their hydrophilic nature, which are made up by the hydrophilic EO blocks. The doxorubicin molecules can then accumulate and sequester within the cancer cell, which increases its intracellular dwelling time and, consequently, facilitates more effective cancer cell killing. This effect has been notably demonstrated with hybrid pluronic micelles (PF127/PF105 and PF127/PF123) coupled with various chemotherapeutics [36,37,38,39,40,41,42]. These hybrid pluronic micelles are, on average, 20 to 30 nm in size, which likely protects them from being pumped out by cancer cells. We were interested in the properties of PF127 micelles since our gel formulation solely consists of PF127. Even though the F127 micelles in our study were considerably smaller with a size of 5 nm, they still exerted strong cytotoxic effects on MC-38 colon cancer cells. This suggests that the small F127 micelles still bear potential for dealing with drug resistance, although this effect might be cancer cell line dependent. When systemically injected, these micelles might possibly reach distant tumors by draining from the leaky tumor vessels, which is referred to as the enhanced permeability and retention effect [41]. This phenomenon has promising implications for the treatment of more distantly located tumors. A possible treatment approach would be to inject the depot more distantly and assess whether there are micelles draining to the distant tumor.

SEM pictures clearly revealed the porous structure of P407 hydrogels. Hydrogel morphology study notably has utility when speculating about mechanisms driving drug release. Doxorubicin is a hydrophilic compound and its release is likely driven by diffusion from the pores. On the other hand, gel degradation can also facilitate drug release. Release and degradation have both been considered in this study. The release kinetics of chemotherapeutic drugs from hydrogels have been reported in different studies. In the study of Mao et al. [43], paclitaxel liposomes were incorporated in P407 hydrogel. P407 hydrogel extended paclitaxel release up to 120 h. In a comparable study, Nie et al. [44] loaded paclitaxel in P407 hydrogels and reported 100% drug release within 14 h. However, drug release was drastically retarded while using a membrane. Here, we found that doxorubicin was released within 120 h using the membrane-less method. A plausible explanation for this discordance is the larger initial gel volume used here (1 mL), leading to slower erosion and consequently longer doxorubicin release. Additionally, larger water volumes can cause the quick dilution of P407 hydrogels. With this information, it can be anticipated that hydrogel drug release kinetics in the body might differ, depending on the route and location of gel administration. Hydrogel dilution is notably pronounced at those sites with more biological fluid, such as the mouth and vagina [45]. Thus, the results of in vitro release studies can vary strongly, depending on the experimental set-up, even for (nearly) similar formulations. Thus, considering an ideal in vitro release study set-up to mimic in vivo situations might give rise to significant discrepancies due to its remoteness from in vivo situations.

To solve these problems, non-invasive imaging could be used as a convenience tool that allows the tracking of fluorescent labelled drugs [46,47,48]. Although in vivo release studies provide valuable information regarding the drug concentration in circulation [8,14], they only provide partial information about where drugs my spread or accumulate after dosing. By taking advantages of the conveniences of non-invasive imaging techniques, we observed how hydrogel ICG depots were retained for at least one week at the site of injection. This indicates that doxorubicin, bearing comparable water solubility and kDa size, is released in a sustained manner from the injection site. Mao et al. [43] loaded P407 liposomal hydrogels (20% P407/5% P188) with paclitaxel and studied the in vivo drug retention using imaging. Interestingly, signals were high until day 3, but diminished after day 4. However, it should be noted that P188 accelerates hydrogel breakdown and it has a considerably lower viscosity than P407 [14,49]. In another paper, P407 hydrogels (22%) were loaded with doxorubicin for a combined chemo-radiotherapeutic regimen. The gels were injected intratumorally and tumors were harvested for imaging. Fluorescence signals in the tumor were peaking at day 1, but diminished after day 2 [20]. It should be noted that, in both studies, depots were injected intratumorally. It cannot be ruled out that the pressure resulting from the rapidly growing tumor results in quicker gel breakdown and, thus, quicker drug release. Furthermore, the tumor has presumably an acidic extracellular environment, which could also promote quicker drug release [42,50]. In our setting, no tumors were present, which might explain the longer hydrogel retainment (seven days minimally). When considering a possible role the tumor has on release rates, it is worth studying the optimal location of injection, e.g., intratumorally or more distantly from growing tumors.

Nonetheless, it becomes evident that concentrating the drug load at the injection site is gaining interest. The cytotoxic drug can be released slowly and locally, thereby preventing high systemic (toxic) peak levels. Recent studies have underscored these prospects by exploiting gemcitabine loaded nanocapsules hydrogels for the local treatment of different tumor types [51,52,53,54]. Taken together, it is conceivable that upcoming research will devise more delivery systems to improve the local delivery of anti-cancer drugs.

Altogether, we report a versatile analysis of a P407–doxorubicin formulation in order to obtain detailed information regarding pharmacological, biological, and imaging parameters. This increases the understanding of hydrogel delivery systems and this knowledge can, for instance, be extended to other studies focusing on the further fine tuning of hydrogels. Tailor-made hydrogels may offer promising prospects for the development of more effective and, above all, safer chemotherapies.

## 4. Materials and Methods

### 4.1. P407 Hydrogel Preparation

The ′cold′ method was deployed to prepare hydrogels [55]. To prepare a 25% poloxamer hydrogel formulation, 2.5 g poloxamer powder (Sigma Aldrich; Steinheim; Germany; molecular weight 9840–14,600 g/mol) was slowly dissolved in 10 mL PBS and then stirred overnight at 4 °C until all of the granules were dissolved. Doxorubicin (Leiden University Medical Center Pharmacy, Leiden, The Netherlands; 50 µg/mL) was then added in the gel.

### 4.2. Time-to-Gelation Assay

Thermosensitive hydrogels undergo reversible thermo-gelation when increasing the temperature above the critical gelation temperature. The time that it takes to reach this point is referred to as time-to-gelation. The time-to-gelation was measured at RT and 37 °C for several P407 concentrations by repeatedly pipetting 250 µL of hydrogel up and down. The timer was stopped upon the pipet tips became clogged.

### 4.3. Morphology of P407 Hydrogel by Scanning Electron Microscopy (SEM)

P407 hydrogels were frozen at −80 °C and then lyophilized. After freeze drying, small pieces of P407 hydrogels were cut, loaded on aluminum stubs, and sputter coated with a platinum layer. P407 hydrogel morphology was examined with scanning electron microscopy (SEM; NanoSEM 200 Microscope, FEI, Shinkawa, Japan). Pore size was determined with ImageJ software (National Institutes of Health, Bethesda, MD, USA).

### 4.4. P407 Hydrogel Degradation and Doxorubicin Release

Doxorubicin release was evaluated while using the ′membrane-less′ release method, as described in previous reports [27,28]. 1 mL of hydrogel mixed with 50 µg doxorubicin was loaded in a 15 mL falcon tube and then stored at 37 °C for 1 min. to induce gelation. Thereafter, 4 mL of 37 °C PBS was carefully layered on the gel layer. At the indicated time-points (2 h, 4 h, 6 h, 8 h, 24 h, 48 h, 72 h, 96 h, and 120 h), 1 mL sample was withdrawn and the volume was replaced with 1 mL fresh PBS. After the final time-point, the samples were diluted and transferred to black 96-wells plates. Doxorubicin fluorescence (600 nm peak) was quantified with the Cytation 5 manager plate reader (BioTek® Instruments, Inc., Winooski, VT, USA). The doxorubicin standard curve was prepared under the same experimental conditions as the samples for release studies. In a separate analysis, it was evident that doxorubicin emission intensity in PBS and P407 solutions differs considerably (Appendix A: Table A1). Briefly, a known amount of doxorubicin was loaded in P407 hydrogel and PBS was layered on top. After one week, the hydrogel was fully degraded and this solution (end concentration 50 µg/mL doxorubicin) was diluted to create the calibration curve. The cumulative release was calculated according to Equation (1):

(1) $$E(\%)=(\frac{V_{E}\sum_{1}^{n-1}C_{i}+V_{0}C_{n}}{m_{0}})\times 100$$

where *E*(%) is the cumulative release, *V_E_* is the withdrawn volume (1 mL), *V*_0_ is the begin volume (4 mL), *C_i_* and *C_n_* are the doxorubicin concentrations, *i* and *n* are the sampling times and *m*_0_ is the total amount of doxorubicin loaded in the hydrogel (50 µg).

Alongside, hydrogel breakdown was measured. First 1 mL of hydrogel was loaded in 15 mL falcon tubes, and the difference in tube weight before and after loading is defined as 100% gel weight. Subsequently, 4 mL of PBS was carefully layered on top. At pre-determined time-points (corresponding to those from the release study), the entire volume was withdrawn and the change in falcon tube weight was measured to calculate the amount of broken down hydrogel.

### 4.5. Dynamic Light Scattering (DLS) Measurements

Various concentrations of P407 (2.5%, 5.0%, 7.5% and 10.0%) were prepared in MilliQ H_2_O (Merck-Millipore, Burlington, MA, USA) to characterize the P407 micelles. Approximately 700 µL of the samples were then transferred to cuvettes and then measured on a Zetasizer (Nano ZS, Malvern Ltd., Malvern, UK).

### 4.6. Cytotoxicity (MTS) Assays

MC-38 tumor cells (kindly provided by Mario Colombo) were cultured in 96-wells plate (5 × 10^3^/well) with titrated amounts of P407 (max concentration 25 mg/mL), doxorubicin (max concentration 500 ng/mL), or a combination of both to assess the cytotoxic effects of P407 and doxorubicin. The cells were cultured in IMDM medium (Lonza, Walkersville, MD, USA) containing Hepes (25 mM). The medium was enriched with 10% fetal calf serum (FCS; Sigma-Aldrich, St. Louis, MO, USA), 2 mM L-Glutamine (Gibco, Paisley, UK), β-mercaptoethanol, and 100 IU/mL penicillin/streptomycin (Gibco, Paisley, UK).

After 24, 48, and 72 h, MTS assay was performed to evaluate cell metabolic activity (as a measure of cell viability). First, old medium was carefully replaced by fresh one and 20 µL MTS reagent (CellTiter 96^®^, Promega, Madison, WI, USA) was added. After 1 h to 4 h, depending on the rate of the enzymatic reaction, a brownish product was yielded, where the optical density (OD) was read at 490 nm using a spectrophotometer. Wells containing medium only were included as background control. The cell viability was calculated according to Equation (2):

(2) $$\%\;Cell\;\mathrm{vibility}=\frac{OD490\;nm\;treated\;cells-OD490\;nm\;background}{OD490\;nm\;untreated\;cells-OD490\;nm\;background}\times 100$$

### 4.7. In Vivo P407 Hydrogel Fluorescence Follow-Up

P407 hydrogel (200 µL) loaded with 10 µM indocyanine green (ICG) was subcutaneously injected in tumor free BALC/c mice. At pre-determined time-points (0, 6, 24, 48, 72, and 168 h), mice were imaged under the IVIS Spectrum small animal imager (PerkinElmer Inc., Waltham, MA, USA) to visualize the 800 nm fluorescence signals. The fur around the injection site was shaved before imaging. The animal experiment was approved by the Dutch Central Committee on Animal Experimentation under license number AVD116002015271 and it was strictly conducted according to the Dutch animal welfare law.

### 4.8. Statistics

All the data and graphs were generated and analyzed using GraphPad Prism 8.0.1 (244) software (La Jolla, CA, USA). The statistical tests performed are parenthetically mentioned in the figure legends. Unless otherwise stated, the data are displayed as mean ± standard deviation (SD). *p* values < 0.05 are regarded as statistically significant.

## Figures and Tables

**Table 1:** Time-to-gelation measurements P407 hydrogel, reported in seconds. The results are shown as means of a duplicate measurement ± standard deviation (SD).

Formulation	Gelation Time (Seconds) at RT (20 °C)	Gelation Time (Seconds) at 37 °C
16% P407	-	-
20% P407	-	47 ± 2.5
25% P407 ^1^	275 ± 5	30 ± 1
25% P407 DOX	264 ± 1.5	29 ± 1.5
30% P407	133 ± 7.5	18 ± 2.5

^1^ Chosen for subsequent experiments.

**Table 2:** Dynamic Light Scattering (DLS) measurements with the polydispersity index (PDI).

Formulation	PDI
2.5% P407	0.390
5.0% P407	0.321
7.5% P407	0.374
10.0% P407	0.597

## Appendix A

**Table A1:** Doxorubicin emission in PBS and P407 breakdown products.

Condition	Measurement 1 (RFU)	Measurement 2 (RFU)	Average RFU (±SD)
Doxorubicin 10 µg/mL in P407	8926	8390	8658 ± 268
Doxorubicin 10 µg/mL in PBS	1178	1209	1194 ± 16

Explanation: Doxorubicin in PBS and doxorubicin released from P407 display different relative fluorescence units (RFU). P407 breakdown products (such as micelles or polymers) might interact with doxorubicin and thereby signals might be amplified. This is also plausible when doxorubicin molecules are densely clustered together in micelles. Considering the fact that doxorubicin is released in the presence of P407 breakdown products, the calibration curves were prepared in the presence of P407. In this way, release samples and calibration curves are measured under the same conditions.
